# Lacerated Wound of the Neck in a Horse

**Published:** 1882-07

**Authors:** 


### X.—LACERATED WOUND OF THE NECK IN A HORSE.

REPORTED BY DRS. WALTON AND M'LELLAN.

A horse was brought to the hospital bleeding profusely from a punctured and lacerated wound located at the junction of the middle and posterior third of the neck.

The animal was one of a pair that had run away and broke the pole of the wagon, one of the sharpened ends of the broken pole causing the wound.

The direction of the wound was upward and forwards, and it was found when examined to contain several slivers from the broken pole. These fragments of wood were imbedded in the adjacent muscle.

*Treatment.*—All foreign bodies were carefully removed, such arteries as could be reached ligated; but plugging and a compress were the principal means by which the hæmorrhage was arrested.

The following day the bandages were removed, and a drainage tube inserted.

In a few days there was a very free discharge of pus.

About the third day considerable fever was noticed, but it readily yielded to treatment.

At the end of the second week the drainage tube was removed, and four weeks later the animal was so near to complete recovery that he was discharged.

COLUMBIA VETERINARY COLLEGE HOSPITAL, NEW YORK CITY, 1882.

